# A Rapid and Highly Efficient Method for the Identification of Soybean Seed Varieties: Hyperspectral Images Combined with Transfer Learning

**DOI:** 10.3390/molecules25010152

**Published:** 2019-12-30

**Authors:** Shaolong Zhu, Jinyu Zhang, Maoni Chao, Xinjuan Xu, Puwen Song, Jinlong Zhang, Zhongwen Huang

**Affiliations:** School of Life Science and Technology, Henan Institute of Science and Technology/Henan Collaborative Innovation Center of Modern Biological Breeding, Xinxiang 453003, China; zsl_94121@126.com (S.Z.); zjy891@126.com (J.Z.);

**Keywords:** hyperspectral image, transfer learning, pretrained network, soybean seed, variety identification

## Abstract

Convolutional neural network (CNN) can be used to quickly identify crop seed varieties. 1200 seeds of ten soybean varieties were selected, hyperspectral images of both the front and the back of the seeds were collected, and the reflectance of soybean was derived from the hyperspectral images. A total of 9600 images were obtained after data augmentation, and the images were divided into a training set, validation set, and test set with a 3:1:1 ratio. Pretrained models (AlexNet, ResNet18, Xception, InceptionV3, DenseNet201, and NASNetLarge) after fine-tuning were used for transfer training. The optimal CNN model for soybean seed variety identification was selected. Furthermore, the traditional machine learning models for soybean seed variety identification were established by using reflectance as input. The results show that the six models all achieved 91% accuracy in the validation set and achieved accuracy values of 90.6%, 94.5%, 95.4%, 95.6%, 96.8%, and 97.2%, respectively, in the test set. This method is better than the identification of soybean seed varieties based on hyperspectral reflectance. The experimental results support a novel method for identifying soybean seeds rapidly and accurately, and this method also provides a good reference for the identification of other crop seeds.

## 1. Introduction

Accurate identification of soybean seed varieties is of great significance for protecting farmers’ interests, ensuring agricultural production, and maintaining order in the seed market. The usual methods for the identification of seed varieties are morphological analysis [1], chemical analysis (random amplified polymorphic DNA (RAPD) and simple sequence repeat (SSR) molecular markers [2,3], protein electrophoresis [4], liquid chromatography [5], spectral analysis [6,7,8], and image analysis [9]. Morphological analysis requires that the appraiser has extensive experience, and the accuracy is low for two or more varieties with little morphological difference [10]. Although the chemical analysis method has a high identification accuracy, it is destructive to the sample, time consuming, and so difficult to operate that nonprofessionals are not competent to perform it [11].

The spectral analysis method reflects the difference in the internal physical structure and the chemical composition of seed varieties through spectral information, which has the advantages of being fast, accurate, and nondestructive [12]; it has been applied to multiple tasks related to seeds, such as the identification of soybean [13,14], rice [6] and maize [15,16,17] seed varieties, the identification of nontransgenic and transgenic seeds [18,19,20], the identification of seed geographical sources [21], the identification of infected germ seeds [22], the identification of infected pest seeds and healthy seeds [23], identification of the year of seeds [24,25], and the determination of tomato [26], soybean [27,28], corn [28], muskmelon [29], cabbage and radish [30] seed vitality. Less research has been performed on image analysis than spectral analysis, and only some plants have been studied through image analysis, such as peppers [31], paddy [32,33], and corn [34]. Some researchers compared spectral analysis and image analysis for the identification of corn seeds, and the results showed that the classification accuracy based on spectral features (95% and 96.2% for each side) is higher than the classification accuracy based on the morphology and texture features (86.8% and 96.6% for each side), while the combination of the two can reach 96.3% and 98.2% discriminant accuracy for each side [7]. Although the spectral analysis and image analysis methods performed well, they still need to go through the processes of reflectance extraction, spectral pretreatment, feature band selection, and morphological and texture feature extraction. In addition, there are problems such as fewer choices of varieties, lower identification accuracy, the reflectance is easily affected by seed relative humidity, and poor portability of the identification models.

Deep learning (DL) is an important artificial intelligence method that enables machines to acquire knowledge from data autonomously [35]. In addition, if the seeds are not severely wet (seed color does not change and seeds do not swell), the relative humidity has no influence on the method of hyperspectral imaging combined deep learning. At present, deep networks have been successfully applied to plant disease identification [36,37,38], drought monitoring [39], land type classification [40], weed detection [41], and other areas of agriculture. To date, there are few reports on the identification of soybean seed varieties by deep learning, and whether it has advantages that is also unknown. Although some studies used deep learning combined with spectral analysis for seed identification [42,43,44], these all used one-dimensional spectra as input, while three-dimensional images contain more information. Moreover, in practical applications, neural networks are usually not trained from scratch for a new task: such an operation is obviously very time consuming. In particular, these models have a large number of parameters that need to be trained and require a very large amount of data. When the amount of data required to construct the model cannot be obtained, the model may be overfit or may fall into a local optimal solution [45,46,47,48]. Using transfer learning to fine-tune the network is faster and easier than randomly training the weights from scratch and does not require many images.

In this paper, after obtaining the hyperspectral images of 9600 soybean seeds from 10 soybean varieties and taking the images as input, pretrained networks, such as AlexNet and ResNet18, were used to carry out transfer training. It was also compared with the traditional machine learning based on spectral reflectance. The aim of this study was to demonstrate that it is feasible and superior to use a deep learning model for seed variety identification based on images as input and to provide a theoretical basis and practical method for the more rapid, accurate, and nondestructive identification of soybean varieties.

## 2. Results and Discussion

### 2.1. Training Progress

**Figure 1:**
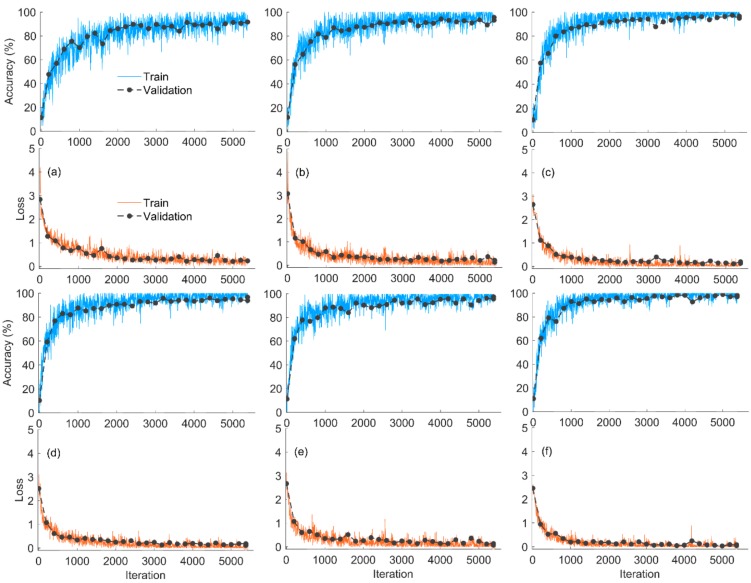
Training progress of the six pretrained models. (**a**) AlexNet; (**b**) ResNet18; (**c**) Xception; (**d**) InceptionV3; (**e**) DenseNet201; and (**f**) NASNetLarge.

The language of deep learning in this article was MATLAB (MATLAB R2019a, The Math Works Inc., Natick, MA, USA), the graphics processing unit (GPU) was a NVIDIA GeForce RTX 2080Ti, and the display memory was 11 GB. Among 0–800 iterations, the accuracy increased and the loss declined rapidly (Figure 1). After 800 iterations, the accuracy of the 6 models all reached 75%, the accuracy increased, and the loss declined slowly. When the training was over, the six models (AlexNet, ResNet18, Xception, InceptionV3, DenseNet201, and NASNetLarge) achieved accuracy values of 91.6%, 95.6%, 96.6%, 96.7%, 97.5%, and 98.2%, respectively, in the validation set. The training times of the six models were 18 min, 117 min, 84 min, 798 min, 455 min, and 1914 min, respectively, and the results showed that the training time is not only related to the depth of the network but also to the other factors (such as the width of the network). From the curve comparison of the six models, the training accuracy of AlexNet fluctuated widely, while NASNetLarge had a small fluctuation range. From AlexNet to NASNetLarge, the number of network layers increased, indicating that the fluctuation range of training accuracy was inversely proportional to the number of layers. Moreover, the larger the number of network layers, the fewer the number of iterations to achieve a stable training accuracy (with 1400 iterations, the accuracy of NASNetLarge reached a high level, and there was little change after 1400 iterations). Comparing the training curve and the validation curve, the model did not exhibit the phenomena of negative transfer, over-fitting, or under-fitting. The results show that transfer training was very successful.

### 2.2. Test Results

**Figure 2:**
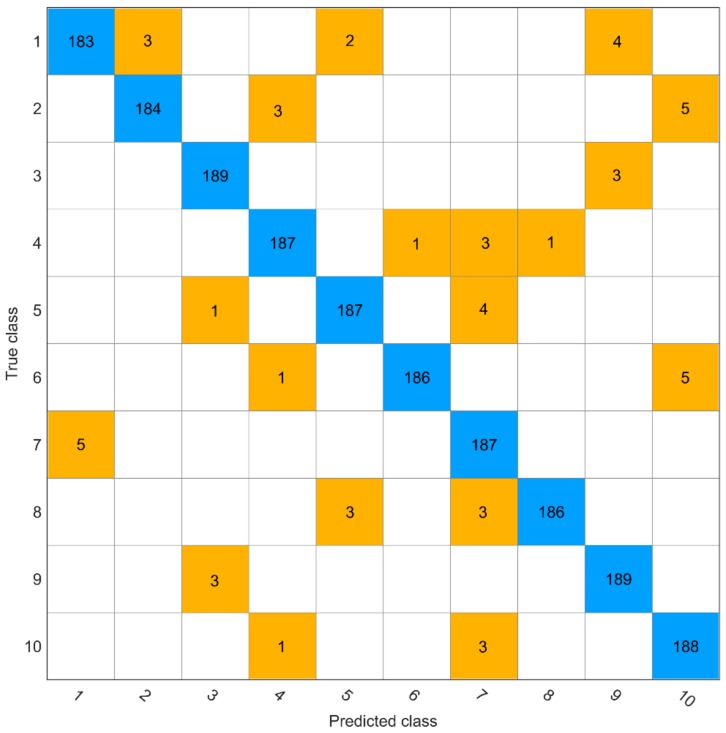
Confusion matrix. (1) Nannong 1606; (2) Shangdou 161; (3) Shangdou 1201; (4) Shangdou 1310; (5) Yudou 18; (**6**) Yudou 22; (7) Yudou 25; (8) Zheng 196; (9) Zheng 3074; and (10) Zheng 9525.

Compared with the training accuracy and validation accuracy, the test accuracy was the most important evaluation index. AlexNet, ResNet18, Xception, InceptionV3, DenseNet201, and NASNetLarge achieved accuracy values of 90.6%, 94.5%, 95.4%, 95.6%, 96.8%, and 97.2%, respectively, in the test set. Of the six models, NASNetLarge performed the best with 54 misjudgments (Figure 2), and among them, Nannong 1606 had the most misjudgments while Shangdou 1201 and Zheng 3074 had the least misjudgments. According to the study, the greater the number of deep neural network layers, the better the performance of the identification models, and the conclusion is the same in other fields [49,50,51]. However, there are also different conclusions [46,52,53,54], as there are several factors that cause this situation, including dataset, network depth, network width, network structure, and parameter settings, and overall, the deep network is indeed slightly better than the shallow network.

### 2.3. Spectral Pretreatment Process

**Figure 3:**
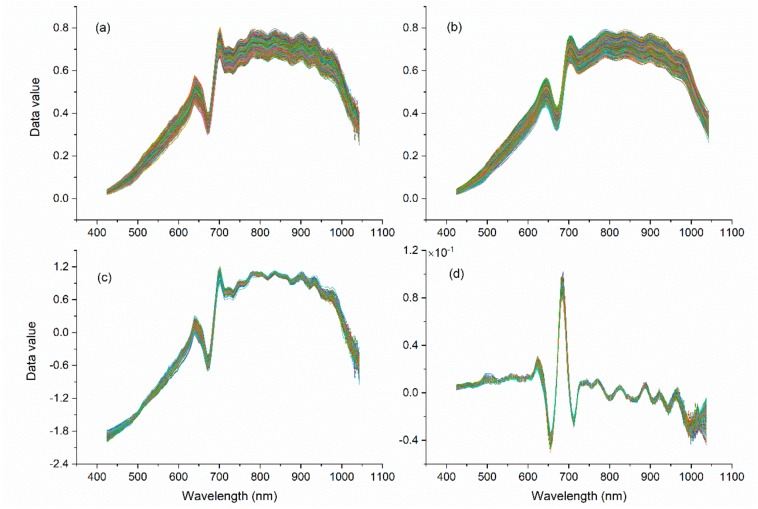
Pretreatment spectrum curves. (**a**) original spectrum (OS) curve; (**b**) Savitzky-Golay (SG); (**c**) standard normal variate (SNV); and (**d**) first derivative (FD).

The pretreatment results are shown in Figure 3. The SG (Figure 3b) results were smoother than those in Figure 3a and eliminated the noise of the original spectrum (OS) at 1000 nm. Since it did not involve the average spectra of all samples, the difference between each spectral curve was still large. SNV needs to be calculated based on the spectral average of all wavelength points in a sample. Therefore, the difference was significantly reduced (Figure 3c) between samples after pretreatment compared with Figure 3a. The geometric meaning of the derivative is the tangent slope of the curve at a certain point, so the derivative can magnify the difference. With FD pretreatment, the spectral differences among different soybeans were mainly in the ranges of 623–638 nm, 649–659 nm, and 675–687 nm (Figure 3d), and these different bands were all within the range of bands with large differences in the original spectra, which indicates that derivative transformation highlighted the characteristic wavelengths.

### 2.4. Identification of Models Using Hyperspectral Reflectance

**Figure 4:**
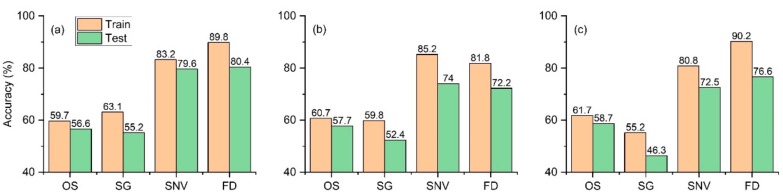
Identification accuracy based on the hyperspectral reflectance of three classifiers with different pretreatments. (**a**) Grid search optimization support vector machine (GS-SVM) classifier; (**b**) ensemble learning (EL) classifier; and (**c**) artificial neural network (ANN) classifier.

Through PCA, the numbers of principal component factors extracted from the three pretreatments were 4, 7, and 24, and the cumulative loads were 97.3%, 85.0%, and 61.3%, respectively. The model based on original spectrum had achieved the highest training accuracy and test accuracy values of 61.7% and 58.7%, respectively. By comparison, it is useful to preprocess the spectral reflectance, and the FD/GS-SVM combination had the highest identification accuracy, with training accuracy and test accuracy values of 89.8% and 80.4%, respectively (Figure 4). The identification model based on the hyperspectral reflectance was sensitive to the pretreatment method, feature extraction method, and classifier. The results obtained by using different combinations were very different. Other studies also reached this conclusion [55,56,57]. There are many steps in the spectrum analysis method. Moreover, as the number of steps increases, the uncertainty of the model will also increase. For example, if the optimal combination of varieties A and B is X, the optimal combination of varieties A and C is Y, and the optimal combination of varieties B and C is Z, then the optimal combination of varieties A, B, and C may not be X, Y, or Z. It is difficult to find an optimal combination with a high identification accuracy and strong portability.

### 2.5. Comparison Analysis

In this study, two identification methods of soybean seed varieties (the hyperspectral image-based deep learning and the hyperspectral reflectance-based machine learning) were compared. The results showed that the worst model (AlexNet with 90.6% test accuracy) of deep learning was better than the best model (FD/GS-SVM with 80.4% test accuracy) of machine learning. In addition, the deep learning method need not to extract the spectral reflectance or perform spectral preprocessing and feature extraction, and there was no need for image cropping or the manual extraction of morphological and texture features of hyperspectral images, which saves a great deal of time. So, the hyperspectral imaging combined deep learning method was superior to the identification model based on hyperspectral reflectance in all respects.

Data diversity is one of the key factors to ensure the generalization ability of the model [37]. Although the study used nearly 10,000 images, the amount of data was still small for 10 soybean varieties. In addition, these models were highly accurate in identifying the 10 soybean varieties, but further verification is needed to determine whether the pretrained models selected for this study will still achieve a high accuracy in the identification of seeds from dozens or hundreds of soybean varieties or whether better CNN models are needed to achieve satisfactory results. The identification model based on hyperspectral images combined with transfer learning is significantly faster than the identification method based on hyperspectral reflectance, but the SOC-710VP imaging spectrometer still needs 35 s to acquire one hyperspectral image. A next step is to determine whether digital cameras or mobile phone cameras can accurately identify images so that this identification method can meet the needs of ordinary people, rather than the needs of professionals. Transfer learning is an important research direction in the field of artificial intelligence in the next few years, and its development provides new research ideas and approaches for the quality and safety detection of agricultural products [58]. The use of machine vision technology combined with deep learning to achieve grain variety detection, grain grading, automated fruit and vegetable variety (origin) detection, and high identification accuracy, etc., and all efforts should be made to increase the research and promotion of this technology.

## 3. Materials and Methods

### 3.1. Materials

The choice of seed variety is the primary consideration in the identification of seed varieties. In terms of material selection, this experiment fully tested the feasibility and ability of hyperspectral images combined with a transfer learning model to identify grain varieties based on seed type, luster, hilum color, 100-seed weight, crude protein content, and crude fat content (Table 1). We selected 10 varieties, which all have yellow seed coats, that are grown on a large scale. One hundred twenty complete, undamaged, spotless seeds per variety were selected and divided into a training set, validation set, and test set with a 3:1:1 ratio. The samples were put in a 38 °C oven for 24 h to ensure the same relative humidity of each variety.

### 3.2. Equipment

**Figure 5:**
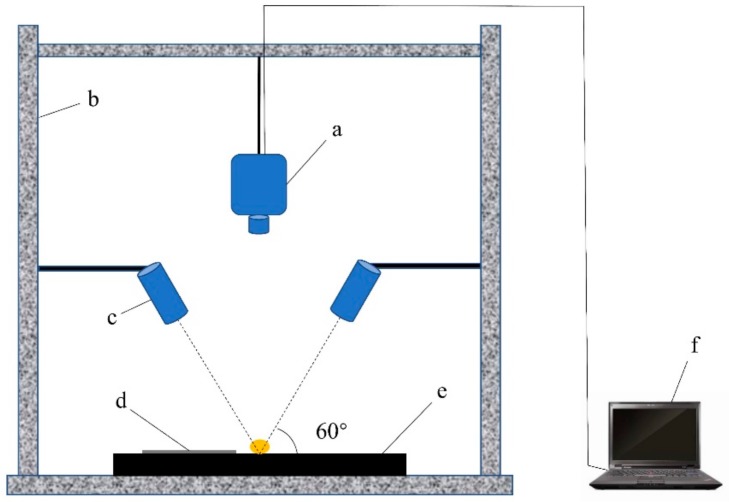
Hyperspectral imaging system. (**a**) Imaging spectrometer; (**b**) darkroom; (**c**) light source; (**d**) standard gray Spectralon panel; (**e**) loading stage; and (**f**) computer.

This study used an SOC-710 portable hyperspectral imaging spectrometer (SOC 710VP, Surface Optics Corporation, San Diego, CA, USA) with a spectral range of 400–1000 nm and a spectral resolution of 4.6875 nm. The light source was two 100 W halogen lamps (Lowel Pro-light, Lower Light Manufacturing Inc., Hauppauge, NY, USA). Other equipment, such as a darkroom, standard gray Spectralon panel and computer, were used. The standard gray Spectralon panel used for reflectance conversion was placed near the seed. The hyperspectral imaging spectrometer lens was placed 30 cm away from the stage, and the incident light of the halogen source was set at an angle of 60° to the stage (Figure 5).

### 3.3. Hyperspectral Image Acquisition

**Figure 6:**
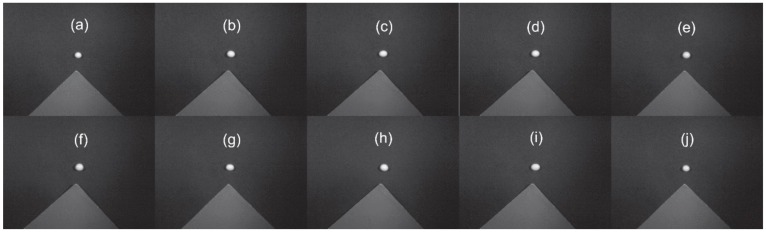
Hyperspectral images of the front of each soybean variety. (**a**) Nannong 1606; (**b**) Shangdou 161; (**c**) Shangdou 1201; (**d**) Shangdou 1310; (**e**) Yudou 18; (**f**) Yudou 22; (**g**) Yudou 25; (**h**) Zheng 196; (**i**) Zheng 3074; and (**j**) Zheng 9525.

SOC 710 Acquisition Software was used to collect images: the integration was set to 20 ms, and the gain was set to 3. First, the lens was covered to obtain black field (dark current) data. Then, the cover was removed to collect the front and back hyperspectral images of the seed (Figure 6). The standard gray Spectralon panel was placed near the seed, which was used for reflectance conversion. Finally, SRAnal 710 (Surface Optics Corporation, San Diego, CA, USA) was used for radiation calibration (the spectrometer manufacturers provided the radiation calibration) and dark current correction.

### 3.4. Image Preprocessing

Because the size of the original images is 696-by-520-by-128 (they are the length, width, and number of bands of the images, respectively), principal component was extracted using principal component analysis (PCA) from the 128 bands. To match the three channels of RGB images, we retained the first three principal components. Blank pixels were added to both sides of the short edge of the images before resizing; otherwise the image underwent deformation. Bicubic interpolation algorithm was used to resize the whole image to match the model input size, as each model requires different input image resolutions (Table 2). Finally, the images were rotated 90°, 180°, and 270°, a total of 9600 images were obtained and the numbers of images in the training set, validation set, and test set were 5760, 1920, and 1920, respectively.

### 3.5. Pretrained Networks

**Figure 7:**
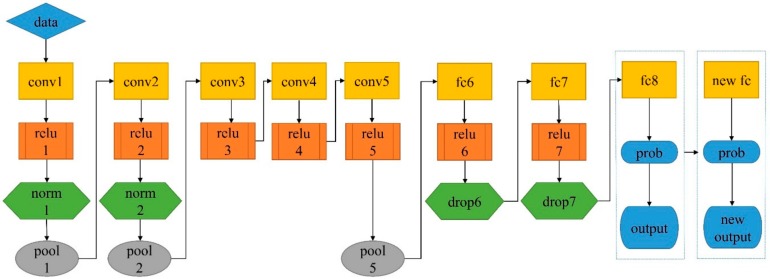
The architecture of AlexNet: conv, relu, norm, pool, fc, drop, and prob are the abbreviations of convolution layer, relu layer, cross channel normalization layer, maxpooling layer, fully connected layer, dropout layer, and softmax layer, respectively.

Six pretrained networks were used to transfer the model parameters. All of these networks were trained on more than one million images of 1000 categories from the ImageNet database and achieved good recognition accuracy. AlexNet [59] is the simplest network model in this study (Figure 7). Transfer learning refers to a learning process that applies a model learned in an old domain (source domain) to a new domain (target domain) by the similarity between the two domains [48]. In this study, the source domain was the ImageNet dataset, and the target domain was soybean hyperspectral images. The type of transfer learning was parameter/model based which was the most widely used. The structure of neural network can be transferred directly, and fine-tune was a good embodiment of parameter/model based transfer learning.

### 3.6. Model Parameter Settings

For classification problems, pretrained models generally consist of an input layer, a convolution layer, a rectified linear unit layer, a pooling layer, a fully connected layer, and an output layer. The number of layers in each model is shown in Table 2. When using transfer learning to solve the problem in this study, fine-tuning only the parameters of the last few layers of the model can achieve the goal of transfer learning. As shown in Figure 7, the AlexNet model was applied to soybean seed identification by fine-tuning only the fully connected layers (fc8) and the output parameters. Similarly, for the other five models, the antepenultimate layer was all fully connected layer, and each model had an output size of 1000. We set the output size to 10 to make the network model suitable for the classification of soybean seed hyperspectral images and to debug the parameters, such as the learning rate for the weights (weight learn rate factor). In the process of debugging parameters, different values affect the accuracy of the identification model, and multiple attempts are required to achieve accurate classifications. The last layer of the 6 networks (classification layer) aimed at 1000 category outputs for the ImageNet database, which were replaced with a new classification layer for retraining in this study. The steps mentioned above were completed with MATLAB’s deep network designer, which was a visual toolbox. The optimization algorithm was “Adam”, which combined Adagrad’s ability to deal with sparse gradients and RMSprop’s ability to deal with non-stationary targets. Transfer training was carried out directly after adjusting the image data set and fine-tuning the network. The other parameters are shown in Table 3.

### 3.7. Comparative Experimental Design

#### 3.7.1. Reflectance Conversion

To verify the superiority of deep learning, the traditional identification methods of soybean seed variety based on reflectance were used. First, the environment for visualizing images (ENVI 5.1, Harris Geospatial Solutions, Inc., Boulder, CO, USA) was used to convert the reflectance of soybean seeds. The region of interest (ROI) of the complete seed image was obtained by an image segmentation algorithm, and the average value of this region was taken as the spectral reflectance. A total of 2400 samples were obtained, and the samples were divided into a training set and test set with a 3:1 ratio. The reflectance of soybean was calculated by using the Equation (1):

(1) $$R=\frac{DN}{DN_{N}}\times R_{N}\;$$

where *R* is the reflectance of soybean, DN is the digital number of soybean, and the digital number is the brightness value of remote sensing image pixels, $DN_{N}$ is the digital number of the standard gray Spectralon panel, and $R_{N}$ is the reflectance of the standard gray Spectralon panel. The $R_{N}$ was obtained by precalibration in the laboratory. DN and $DN_{N}$ were measured in this experiment.

#### 3.7.2. Reflectance Preprocessing

The spectral reflectance was pretreated by Savitzky-Golay smoothing (SG), the standard normal variate (SNV), and first derivative (FD). SG performs polynomial least squares fit on the data in a moving window; the number of window points was set to 7, and the polynomial order was set to 2. The SNV algorithm processed each spectrum, and its calculation in essence was a standard normalization of the original spectral data; the SNV was calculated by using the Equation (2):

(2) $$R_{SNV}=\frac{R-\bar{R}}{\sqrt{\frac{\sum_{i=1}^{p}\left(R_{i}-\bar{R}\right)}{p-1}}}\;\;\;\;\;$$

where R is the original spectrum of a sample, $\bar{R}$ is the spectral average of all the wavelength points in a sample, and i = 1, 2, …, p, p is the number of wavelength points.

The geometric meaning of the derivative is the slope of the tangent of the curve at a certain point, and the derivative of waveband λ was calculated by using the Equation (3):

(3) $$FD_{\left(\lambda \right)}=\frac{R_{\left(\lambda +1\right)}-R_{\left(\lambda -1\right)}}{\left(\lambda +1\right)-\left(\lambda -1\right)}\;\;\;$$

where $R_{\left(\lambda +1\right)}$ is the reflectance at the next waveband of λ, $R_{\left(\lambda -1\right)}$ is the reflectance at the last waveband of λ, $\left(\lambda +1\right)$ is the wavelength of the next waveband of λ, $\left(\lambda -1\right)$ is the wavelength of the last waveband of λ.

#### 3.7.3. Principal Component Extraction

The principal component was extracted using PCA. PCA is a statistical analysis method that transforms many variables into a few principal components by dimension reduction technology [21]. It is one of the most common methods to solve redundant or overlapping information. The characteristic values were calculated, and a characteristic value less than 1 indicates that the principal component is not as powerful as the direct use of the original variable. So, principal components with characteristic values less than 1 were removed.

#### 3.7.4. Classifier Selection

Grid search optimization support vector machine (GS-SVM), ensemble learning (EL), and artificial neural network (ANN) classifiers were used for the identification analysis, and the corresponding parameters are shown in Table 4.

### 3.8. Technical Route

**Figure 8:**
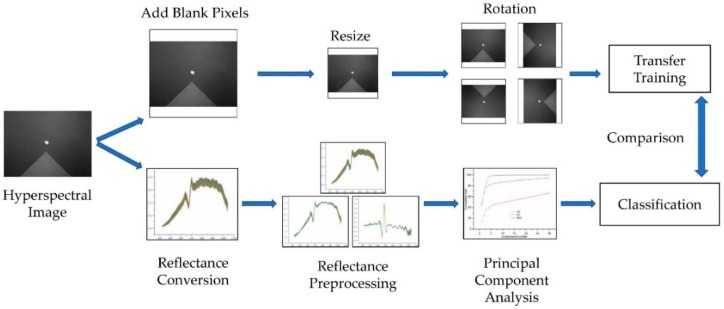
Technical route.

The technical route of this research is shown in Figure 8.

## 4. Conclusions

In this study, 9600 hyperspectral images of 10 soybean varieties were collected, and 6 pretrained networks, such as AlexNet, were used for transfer training to verify the feasibility of the method for identifying soybean seed varieties. The results show that the accurate identification of soybean seed varieties can be realized based on hyperspectral images combined with transfer learning. Moreover, the method based on a hyperspectral image combined with transfer learning has obvious advantages over the method based on the hyperspectral reflectance in terms of the time, operational difficulty, and identification accuracy. In future research, the number of seed varieties and images will be further increased to test the performance of these models, and we hope that these methods can be applied in the seed market.

## Figures and Tables

**Table 1:** Soybean seed quality.

Variety	Seed Type	Luster	Hilum Color	100-Seed Weight (g)	Crude Protein (%)	Crude Fat (%)
Nannong 1606	circular	yes	brown	15.4	36.0	19.7
Shangdou 161	circular	yes	brown	21.6	35.6	19.6
Shangdou 1201	oval	yes	brown	19.1	43.1	20.2
Shangdou 1310	oval	weak	pale brown	18.0	42.1	20.5
Yudou 18	circular	yes	brown	16.8	44.5	18.8
Yudou 22	circular	yes	pale brown	19.3	46.5	18.9
Yudou 25	circular	yes	brown	18.4	46.3	17.1
Zheng 196	circular	weak	pale brown	17.4	40.7	19.5
Zheng 3074	flat oval	weak	pale brown	19.7	40.9	17.1
Zheng 9525	circular	yes	pale brown	21.7	45.0	17.7

**Table 2:** The pretrained models with properties.

Network	Image Input Size	Layers	Network	Image Input Size	Layers
AlexNet	227-by-227-by-3	25	InceptionV3	229-by-229-by-3	316
ResNet18	224-by-224-by-3	72	DenseNet201	224-by-224-by-3	709
Xception	229-by-229-by-3	171	NASNetLarge	331-by-331-by-3	1244

**Table 3:** Parameter settings of all models.

Parameters	Values	Parameters	Values
Momentum	0.9	Max epochs	10
Initial learn rate	0.0001	Mini batch size	10
Initial learn schedule	Piecewise	Shuffle	Every-epoch
Learn rate drop period	10	Validation frequency	200
Learn rate drop factor	0.1	Sequence length	Longest
L2regularization	0.0001	Gradient threshold method	Global-l2norm

**Table 4:** Parameter settings of classifiers.

Classifiers	Parameters	Values
GS-SVM	Kernel function	Linear kernel
	Grid c/g bound	−8–8
	Grid c/g step	0.5
EL	Ensemble method	AdaBoost
	Learning rate	0.1
	Number of learners	30
ANN	Type of neural network	Back propagation
	Number of hidden neurons	15
	Training function	Traingdm
